# Alpha-amylase and Alpha-glucosidase enzymes inhibition and antioxidant potential of selected medicinal plants used as anti-diabetes by Sundanese community in West Java, Indonesia

**DOI:** 10.1186/s12906-025-05144-x

**Published:** 2025-11-14

**Authors:** Raden Maya Febriyanti, Raden Bayu Indradi, Intan Timur Maisyarah, Yoppi Iskandar, Raini Diah Susanti, Dwintha Lestari

**Affiliations:** 1 Faculty of Pharmacy, Universitas Padjadjaran, Bandung, Indonesia; 2 Faculty of Nursing, Universitas Padjadjaran, Bandung, Indonesia; 3 Pharmacy Study Program, Universitas Muhammadiyah Bandung, Bandung, Indonesia

**Keywords:** In vitro *anti-diabetes*, Medicinal plants, Traditional medicine, Phenol, And flavonoid, Principal-component analysis

## Abstract

Plants provide an excellent alternative for managing diabetes and are widely utilized in many developing countries including Indonesia. This study aimed to provide comprehensive scientific information on the α-amylase and α-glucosidase inhibitory of the selected medicinal plants used by Sundanese community in West Java. Twelve medicinal plants were extracted individually in ethanol and their total phenolic content (TPC) and total flavonoid content (TFC) were quantified by Folin–Ciocalteu and AlCl₃ colorimetric assays, respectively. Concentration-dependent inhibition of α-amylase and α-glucosidase, as well as DPPH scavenging, were measured in 96-well plates. IC₅₀ values were obtained by non-linear regression. Data were compared by one-way ANOVA (*P* < 0.001). Autoscaled principal-component analysis (PCA) explored multivariate relationships among TPC, TFC and the bioactivity variables. Among the twelve selected medicinal plants, TPC and TFC varied 20.3–61.9 mgGAE/g and 15.0–64.9 mg/QE g, respectively. *S. mahagoni* seeds possessed the highest TPC, whereas *A. muricata* leaves led in TFC. Only *S. mahagoni* (IC₅₀ = 214 ± 4 µg/mL) and *M. charantia* (274 ± 5 µg/mL) reached 50% α-amylase inhibition. *P. angulata* provided the sole measurable α-glucosidase IC₅₀ (438 µg/mL). Furthermore, *S. mahagoni* exhibited the strongest DPPH scavenging (IC₅₀ = 138.6 µg/mL). PCA explained 69.9% of variance with PC1 (39.5%) loaded positively on α-amylase inhibition (+ 0.83) and TPC (+ 0.81) and PC2 (30.4%) associated with α-glucosidase inhibition (+ 0.81) and TFC (+ 0.62). These data validate Sundanese ethnomedicine and prioritize *S.mahagoni* and *P.angulata* for bioactivity-guided isolation and in vivo antidiabetic evaluation.

**Supplementary Information:**

The online version contains supplementary material available at 10.1186/s12906-025-05144-x.

## Introduction

Diabetes mellitus (DM) is a chronic metabolic disorder marked by persistent hyper-glycaemia. It remains one of the most prevalent global health problems, accounting for 90% of the cases of diabetes, with a mortality incidence of 4.2 million people worldwide [1]. According to the 2021 International Diabetes Federation (IDF) report, approximately 537 million adults are living with DM. The number is expected to increase in 2030 to 643 million and 783 million in 2045 [2]. Regionally, Southeast Asia ranks third, with a DM incidence of 11.3%. Furthermore, according to worldwide data, the incidence of DM in Indonesia is the 7th highest, with 10.7% of the total population affected after China, the USA, India, Russia, Brazil, and Mexico, making Indonesia the only country in Southeast Asia that contributes massively to the global prevalence of DM [3–5].

Diabetes mellitus can lead to severe complications, including nephropathy, neuropathy, retinopathy, and cardiovascular disease. Under these conditions, carbohydrate homeostasis and lipid metabolism are disrupted due to abnormalities in the production, secretion, or action of insulin [6, 7]. One pharmacological strategy to blunt post-prandial glucose excursions is to block gastrointestinal digestion of starch by inhibiting the key carbohydrase enzymes α-amylase and α-glucosidase [8]. Currently, drugs such as acarbose, voglibose, and miglitol, which inhibit α-glucosidase and α-amylase, are widely used as oral hypoglycemic agents. However, these drugs can cause undesirable side effects, such as bloating, abdominal discomfort, diarrhea, and flatulence [9].

Hyper-glycaemia is further exacerbated by oxidative stress, a state in which reactive oxygen species (ROS) overwhelm endogenous antioxidant defences. Excessive ROS simultaneously worsens insulin resistance and impairs pancreatic β-cell function, thereby accelerating diabetic pathology [10, 11]. In diabetic patients, oxidative stress contributes to changes in two primary mechanisms: insulin resistance and impaired insulin secretion [6, 12]. Medicinal plants with high antioxidant potential can mitigate oxidative stress, thereby providing a protective effect against diabetes-induced complications [13]. Phenolics and flavonoids, abundant in many medicinal plants, scavenge ROS, inhibit lipid peroxidation, and modulate endogenous antioxidant defense systems such as superoxide dismutase, catalase and glutathione peroxidase [14, 15]. Studies have demonstrated that flavonoid-rich plant extracts can reduce oxidative damage in diabetic models, thereby preserving pancreatic β-cell function and improving insulin secretion [16].

Indonesia is a tropical region with very high biodiversity and is a source of raw materials for medicines. Furthermore, Indonesia is also known as the country with the largest ethnic groups and cultures in the world [14, 17]. The Sundanese ethnic group, the second largest ethnic group in Indonesia, considered West Java as their homeland and called most of the West Java, *Tatar Sunda*. The Sundanese community in West Java has a long history of utilizing medicinal plants for managing DM [15–17]. Our previous ethnomedicinal study documented the extensive use of medicinal plants by the Sundanese community and identified most frequently used plant species for managing DM [16]. A growing body of in-vitro research has shown that many Indonesian botanicals suppress α-amylase and α-glucosidase activity, the key enzymatic targets for post-prandial glycaemic control [12, 18–20]. Building on this foundation of indigenous knowledge, the present study (i) quantifies the total phenolic (TPC) and flavonoid (TFC) contents of the twelve most-cited species, (ii) measures their inhibitory potency against porcine pancreatic α-amylase and yeast α-glucosidase, (iii) determines their free-radical-scavenging capacity via the DPPH assay, and (iv) integrates these variables through principal-component analysis (PCA). This multivariate approach aims to pinpoint extracts that unite dual enzyme inhibition with strong antioxidant activity, providing culturally consonant, cost-effective leads for future antidiabetic drug development.

## Materials and methods

### Chemicals and reagents

*An α-Amylase Inhibitor Screening Kit* (Abcam) containing the substrate p-nitrophenyl-α-glucopyranoside (PNPG), yeast α-glucosidase, diphenyl-1-picrylhydrazyl (DPPH), and soluble starch (extrapure) was purchased from Sigma‒Aldrich Co., Acarbose (Kimia Farma, tbk.) was purchased from a local pharmacy. Other chemicals, solvents, and reagents were of analytical grade and provided by the Laboratory on Natural Product, Faculty of Pharmacy, Universitas Padjadjaran, Indonesia.

### Instruments

Absorbance measurements were taken with a microplate reader (Infinite M200 Pro, Tecan, Männedorf, Switzerland) and a UV-visible spectrophotometer (SPECORD 205, Analytik Jena, Jena, Germany).

### Plant materials

In this study, twelve of the most cited plants from ethnopharmacological data were selected, namely, *Annona muricata* L., *Moringa oleifera* Lam., *Physalis angulata* L., *Momordica charantia* L., *Swietenia mahagoni* (L.) Jacq., *Muntingia calabura* L., *Centella asiatica* (L.) Urb., *Smallanthus sonchifolius* (Peopp.) H. Rob., *Orthosiphon aristatus* (Blume) Miq., *Persea americana* Mill., *Chromolaena odorata* (L.) R.M. King & H. Rob., and *Piper ornatum* N.E. Br. All plant materials were collected in West Java, Indonesia, specifically from the private gardens of participating traditional healers during our ethnobotanical fieldwork, after obtaining the landowners’ permission and informed consent under an approved ethics protocol for conducting ethnobotanical study. These plant materials were formally identified by Drs. Joko Kusmoro, M.P., at the Herbarium Jatinangoriense, the Biosystematic and Molecular Laboratory, Universitas Padjadjaran. To ensure the reproducibility of our findings, a voucher specimen of each plant studied was deposited at the publicly available herbarium Herbarium Jatinangoriense, Universitas Padjadjaran, Indonesia (Voucher specimen number: 372–383).

### Extraction and determination of water content

**Fig. 1:**
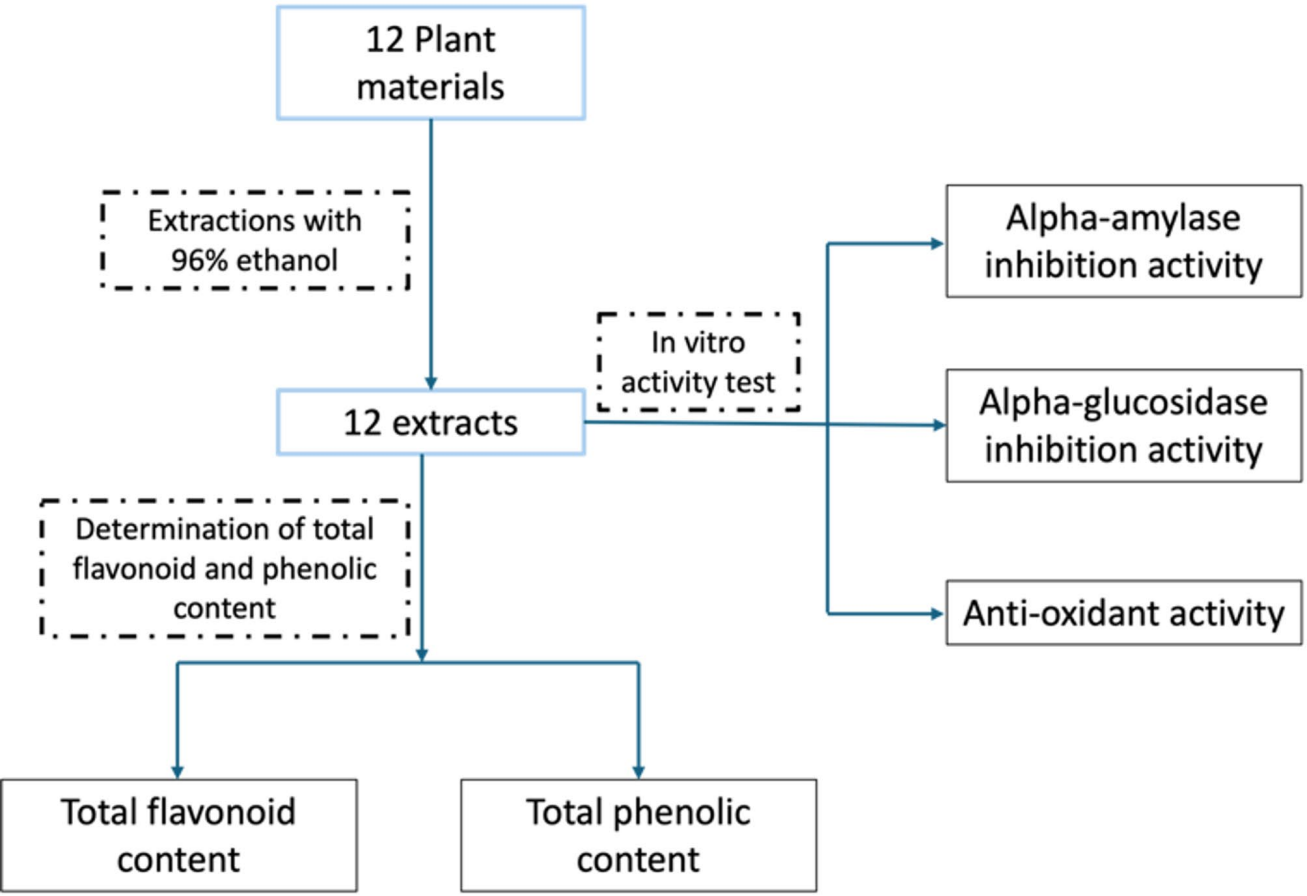
Diagram of methods

The plant materials used in this study were selected based on their traditional medicinal applications and included various parts such as leaves (*A. muricata*, *M. oleifera*, *P. angulata*, *M. charantia*, *S. sonchifolius*, *P. americana*, *C. odorata*, and *P. ornatum*), seeds (*S. mahagoni*), fruits (*M. calabura*), and herbs (*C. asiatica* and *O. aristatus*) as previously reported in our study [16]. All plant materials were shade-dried to preserve their bioactive compounds, finely powdered, and then extracted individually using the maceration method with 96% ethanol. The maceration process involved soaking each powdered plant material in ethanol for three consecutive cycles of 24 h each. The extracts were filtered and concentrated under reduced pressure at 40 °C using a rotary vacuum evaporator. The water content of the extract was determined using the toluene distillation. Several analyses were subsequently performed on the obtained extracts, as shown in Fig. 1.

### Determination of total phenolic content (TPC)

The total phenolic concentration in these extracts was quantified by Folin-Ciocalteu reagent (FCR) using the method conducted by Ghaffari et al. [21]. A mixture of the extract was prepared in ethanol. The resulting mixture was incubated at 45 °C for 15 min, after which the absorbance was measured at 765 nm. A standard curve was prepared by mixing a gallic acid methanol solution with 1 mL of 10% Folin–Ciocalteu reagent and 0.8 mL of 0.075 mg/mL Na_2_CO_3_ solution. The experiments were conducted in triplicate, and the results are presented as the mean values with SDs. The total phenolic content is expressed as milligrams of gallic acid equivalents per gram of dry sample (mgGAE/g) and was calculated using the standard curve of gallic acid.

### Determination of total flavonoid content (TFC)

The amount of total flavonoids in the plant extracts was determined using the AlCl_3_ colorimetric assay Briefly, 1 mL of 2% AlCl_3_ in methanol p.a. (v/v) was added to 1 mL of the extract solution at concentration range 10–200 µg/mL. Following a one-hour incubation at room temperature, a yellow color formed from the reaction was quantitatively measured at wavelength 420 nm using a UV‒Vis spectrophotometer (Analytic Jena – SPECORD 205). The total flavonoid content was determined by constructing a standard curve using quercetin as the reference compound following the same procedure described for the total phenolic content analysis. The experiments were conducted in triplicate, and the results are reported as the mean values with standard deviations. The total flavonoid content in the extracts was calculated and expressed as milligrams of quercetin equivalents per gram of extract (mgQE/g).

### α-Amylase inhibition assay

The α-amylase activity inhibition test was carried out by following the method of Alqahtani et al. [6]. Extract solutions with a concentration of 1.000 µg/mL were prepared using 1% dimethyl sulfoxide (DMSO) and diluted in water for injection. The stock solution was subsequently diluted to concentrations of 500, 250, and 125 µg/mL. Acarbose was used as a positive control in the experiment to validate the assay methods for α-amylase and α-glucosidase inhibition. However, for a more relevant comparison, a commercially marketed herbal medicine for diabetes, containing a combination of active fractions of *Cinnamomum burmani* and *Lagerstroemia speciosa* 100 mg (hereinafter referred to as the Standard Herbal), was selected. The inclusion of the Standard Herbal allows for a comparison within the same category of herbal-based treatments, which are more similar in nature to the tested plant extracts. Then, 100 µL of extracts, acarbose, Standard Herbal and 100 µL of α-amylase were incubated at 25 °C for 10 min. After preincubation, 100 µL of 1% starch solution was added to each well and incubated for 15 min. The reaction was stopped with 10 µL of dinitrosalicylic acid. The absorbance (at a wavelength of 540 nm) was measured using a microplate reader (Infinite M200 Pro TECAN). Mean values were obtained from triplicate experiments. The control samples were prepared without any plant extracts or drugs. The percent inhibition of amylase activity was calculated using the following formula (Eq. 1) and the IC_50_ values were determined graphically using GraphPad Prism 10.

$$\:\varvec{\%}\:\mathbf{I}\mathbf{n}\mathbf{h}\mathbf{i}\mathbf{b}\mathbf{i}\mathbf{t}\mathbf{i}\mathbf{o}\mathbf{n}=\:\frac{\mathbf{A}\mathbf{b}\mathbf{s}.\mathbf{c}\mathbf{o}\mathbf{n}\mathbf{t}\mathbf{r}\mathbf{o}\mathbf{l}-\mathbf{a}\mathbf{b}\mathbf{s}.\:\mathbf{s}\mathbf{a}\mathbf{m}\mathbf{p}\mathbf{l}\mathbf{e}}{\mathbf{A}\mathbf{b}\mathbf{s}.\mathbf{c}\mathbf{o}\mathbf{n}\mathbf{t}\mathbf{r}\mathbf{o}\mathbf{l}}\:\times\:\:100$$

### α-Glucosidase inhibition assay

The α-glucosidase inhibition assay was carried out following the methods of Alqahtani et al. [6]. Extracts were prepared as described for the α-amylase inhibition assay. Eighty microliters of extract at concentrations 125, 250, and 500 µg/mL was mixed with 20 µL of α-glucosidase and incubated at 37 °C for 10 min. Then, 50 µL of 5 mM p-nitrophenyl-α-D-glucopyranoside (pNPG) was added to the mixture to initiate the reaction. The reaction mixture was incubated at 37 °C for 60 min, and the reaction was terminated by adding 2.5 mL of 0.1 M Na_2_CO_3_. α-Glucosidase activity was determined by measuring the absorbance at 400 nm wavelength using a microplate reader (Infinite M200 Pro TECAN). The inhibition of α-glucosidase activity was calculated as a percentage of the total enzyme activity using Eq. 1. The IC_50_ values were determined graphically using GraphPad Prism 10.

### DPPH radical scavenging activity

DPPH radical scavenging activity was determined following the method of Alqahtani et al. [6], with slight modifications. The test was conducted by preparing 6 different concentration series of sample solutions, namely 31.25, 62.5, 125, 250, 500, and 1000 µg/mL. The test concentrations were varied through stepwise dilution steps carried out in a 96-well plate. For the control sample, ethanol was added in place of the test extracts. The absorbance was measured at 520 nm after 30 min of incubation. The percent inhibition was calculated using Eq. 1. The IC_50_ values were determined graphically using GraphPad Prism 10.

### Statistical analysis

All the experiments were performed in triplicate, and the results are expressed as the mean ± SD. The data were analyzed by one-way ANOVA (*p* < 0.001) compared to the negative control. To explore multivariate relationships among total phenolic content (TPC), total flavonoid content (TFC) and the three bioactivity variables (α-amylase inhibition, α-glucosidase inhibition, and DPPH IC₅₀), a principal component analysis (PCA) was performed using IBM SPSS Statistics v30. Variables were autoscaled to unit variance prior to analysis. Components with eigenvalues > 1 were retained, and loadings ≥ |0.50| were considered significant.

## Results

### Extraction yield, moisture content, total phenolic content (TPC), and total flavonoid content (TFC)

**Fig. 2:**
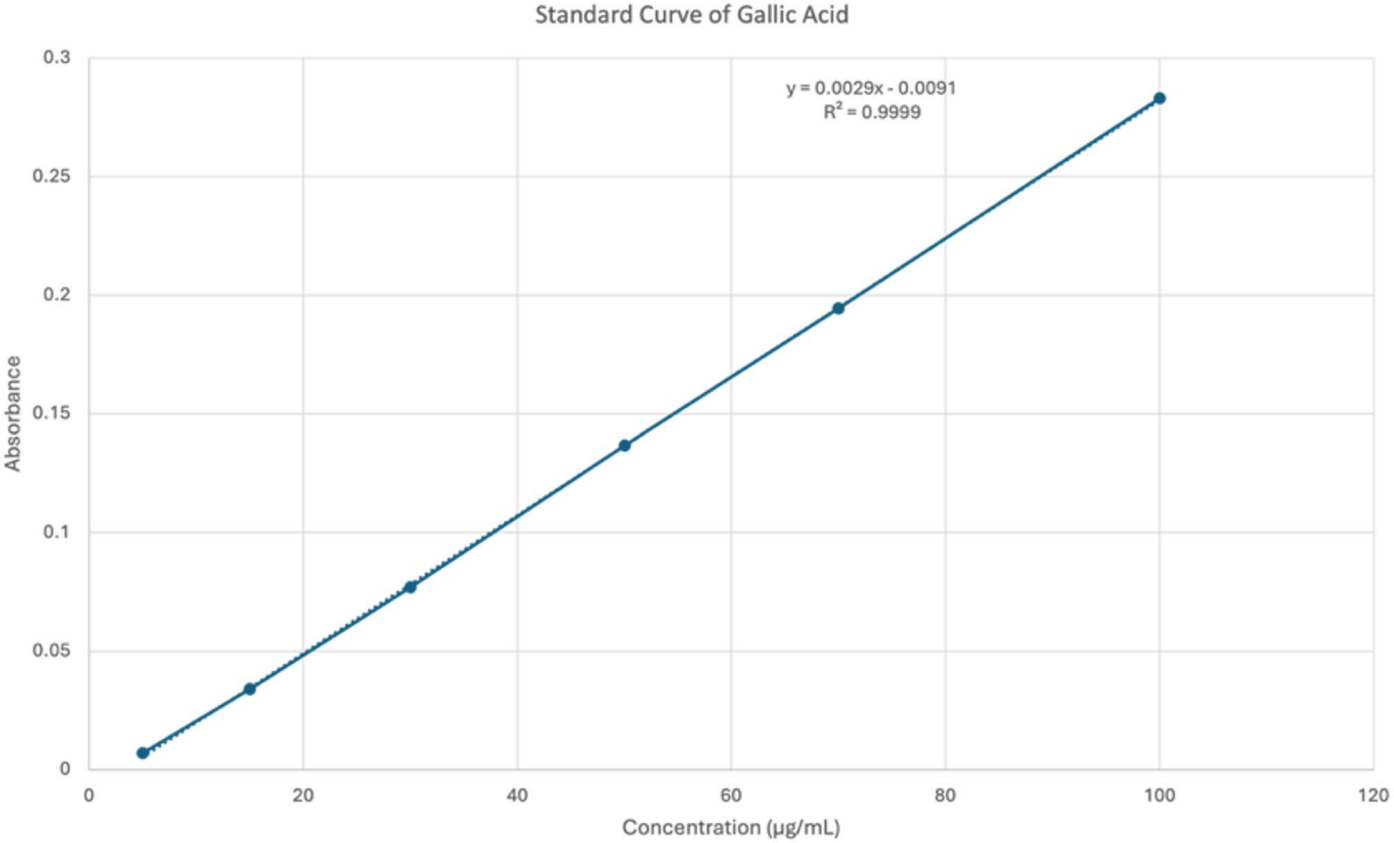
Standard curve of gallic acid

**Fig. 3:**
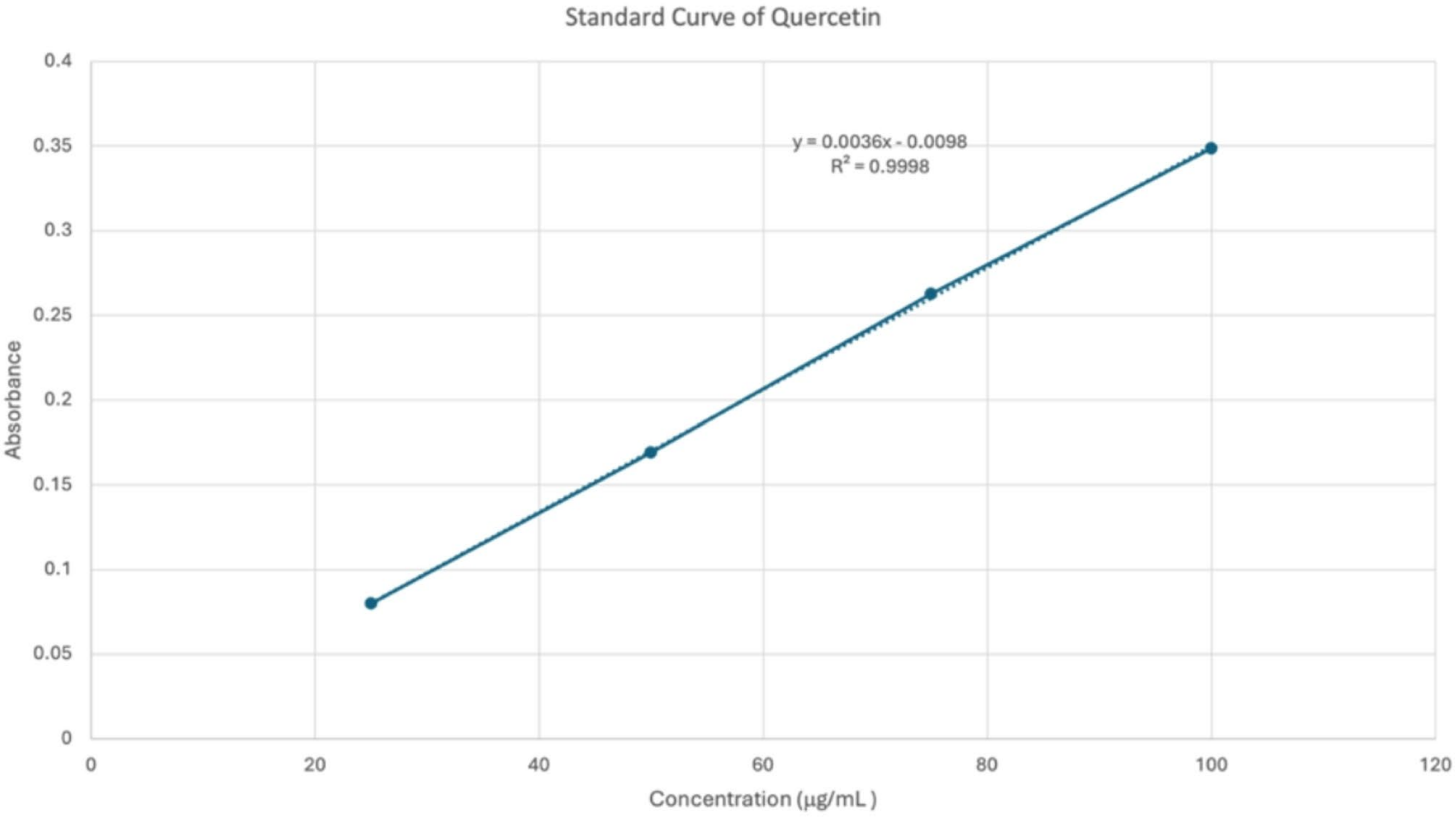
Standard curve of quercetin

Extraction yields, water content, total phenolic content, and total flavonoid content are summarised in Table 1. Extract yields ranged from 11.32% (*C. asiatica*) to 29.67% (*P. angulata*). Total phenolic content (TPC) and total flavonoid content (TFC) were quantified using standard curves of gallic acid and quercetin, respectively (Figs. 2 and 3). The highest phenolic content was found in *S. mahagoni* seeds (61.93 mg GAE/g), while the highest flavonoid content was in *A. muricata* leaves (64.87 mg QE/g). Notably, *S. mahagoni* also exhibited a high TFC (60.70 mg QE/g). Water content was uniformly below 10% across all samples, indicating proper drying procedures and preservation of bioactive constituents.

**Table 1:** Extraction yields, water content, total phenolic content, and total flavonoid content of extracts

Medicinal plants	Parameter			
	Extract yield (%)	Water content (%)	Total Phenolic Content (mgGAE/g)	Total Flavonoid Content (mgQE/g)
*A. muricata*	27.20	8.63 ± 1.65	29.67 ± 0.28	64.87 ± 0.63
*M. oleifera*	12.25	7.31 ± 0.75	23.45 ± 1.13	15.03 ± 0.11
*P. angulata*	29.67	8.44 ± 1.31	29.43 ± 1.42	47.41 ± 1.40
*M. charantia*	18.87	9.35 ± 1.89	28.16 ± 0.94	26.63 ± 1.43
*S. mahagoni*	17.15	6.95 ± 0.86	61.93 ± 0.44	60.70 ± 1.33
*M. calabura*	18.97	8.63 ± 1.56	54.60 ± 1.63	35.06 ± 0.54
*C. asiatica*	11.32	9.42 ± 2.03	39.66 ± 0.74	17.07 ± 1.29
*S. sonchifolius*	16.48	6.97 ± 0.85	35.11 ± 0.45	28.45 ± 0.42
*O. aristatus*	12.43	8.79 ± 1.78	22.93 ± 0.28	33.91 ± 0.35
*P. americana*	28.32	8.57 ± 0.54	43.68 ± 1.41	60.92 ± 1.41
*C. odorata*	26.43	6.46 ± 1.12	20.31 ± 1.51	49.04 ± 1.51
*P. ornatum*	19.47	7.52 ± 1.42	55.36 ± 1.43	27.78 ± 0.54

Crude extract yields ranged from 11.32% (*C. asiatica*) to 29.67% (*P. angulata*). *S. mahagoni* exhibited the highest TPC (61.93 mgGAE/g) and one of the highest TFC values (60.70 mg QE/g), whereas *P. americana* showed a flavonoid-rich but only moderately phenolic profile. Moisture contents were uniformly < 10%, indicating adequate drying.

### α-Amylase inhibition

**Fig. 4:**
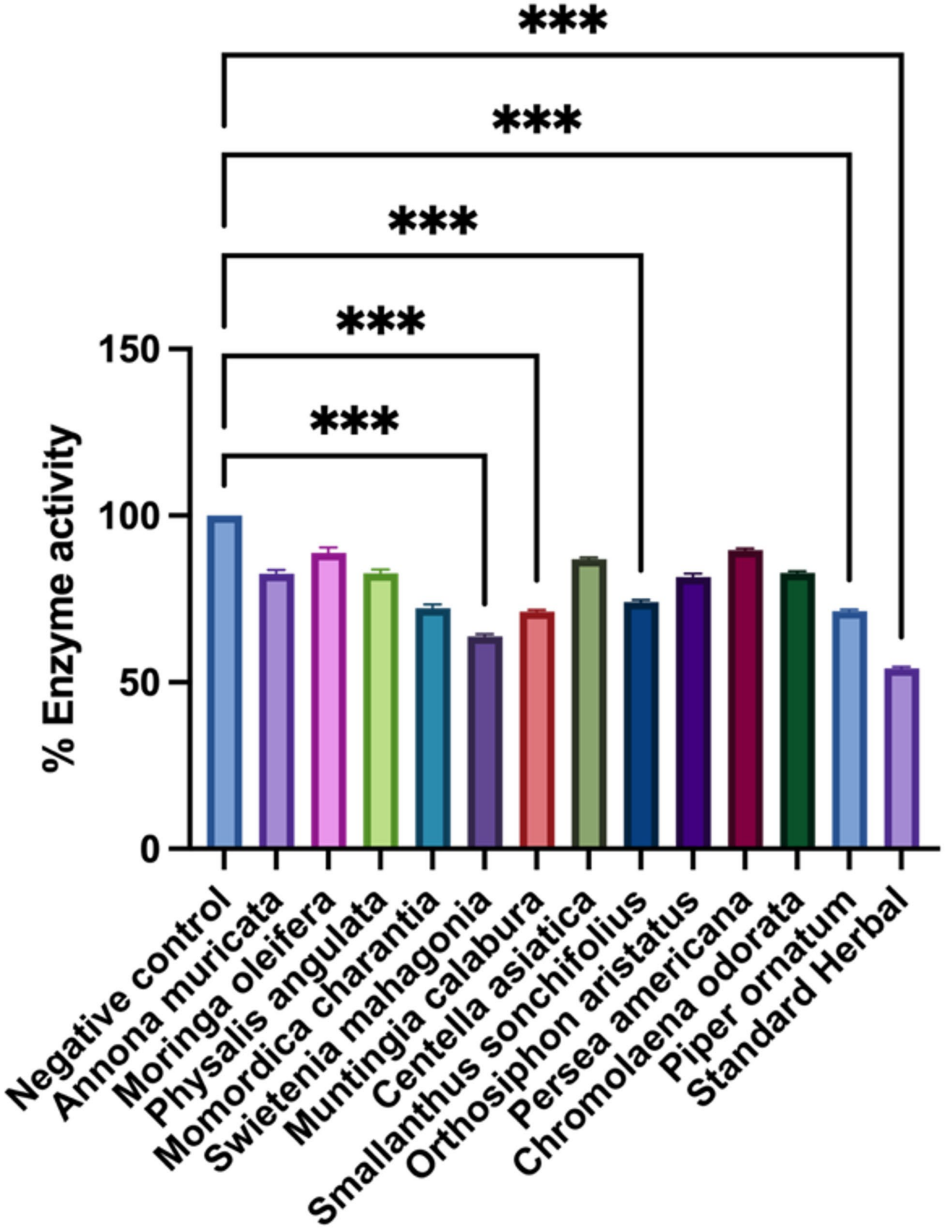
% inhibition of α-amylase activity in samples treated with 125 µg/ml. The data are expressed as the mean ± S.D. Asterisks (***) denotes significantly different (*P* < 0.001) from the negative control (Blank)

The α-amylase inhibitory activity of extracts is shown in Table 2. At the highest tested concentration (500 µg/mL), only *S. mahagoni* (77.52%), *M. charantia* (68.04%), and *P. ornatum* (54.74%) surpassed 50% inhibition, yielding IC₅₀ values of 214 ± 4 µg/mL, 274 ± 5 µg/mL, and 428 ± 7 µg/mL, respectively. Extracts showing < 50% inhibition at 500 µg/mL had IC₅₀ values designated as > 500 µg/mL. At 125 µg/mL, *S. mahagoni* showed the highest inhibition (36.15%), significantly greater than the negative control (*p* < 0.001) (Fig. 4).

**Table 2:** Inhibition of α-amylase activity by extracts at various concentrations

Medicinal plants	Enzyme inhibition at various concentration (%)			Calculated IC_50_ (µg/mL)
	125 µg/mL	250 µg/mL	500 µg/mL	
*A. muricata*	17.44 ± 1.085	18.61 ± 1.06	28.89 ± 0.63	> 500
*M. oleifera*	11.25 ± 1.682	28.21 ± 1.07	31.69 ± 0.60	> 500
*P. angulata*	17.31 ± 1.090	29.14 ± 1.10	35.95 ± 0.57	> 500
*M. charantia*	27.75 ± 1.042	48.06 ± 0.98	68.04 ± 0.67	274 ± 5
*S. mahagoni*	36.15 ± 0.580	55.70 ± 0.98	77.52 ± 0.51	214 ± 4
*M. calabura*	28.79 ± 0.525	40.31 ± 0.54	45.04 ± 1.14	> 500
*C. asiatica*	13.08 ± 0.561	18.46 ± 1.07	29.28 ± 0.53	> 500
*S. sonchifolius*	25.93 ± 0.650	31.06 ± 0.98	35.39 ± 0.57	> 500
*O. aristatus*	18.38 ± 0.982	30.10 ± 1.45	47.78 ± 0.59	> 500
*P. americana*	10.36 ± 0.560	22.96 ± 1.60	34.04 ± 0.51	> 500
*C. odorata*	17.21 ± 0.545	19.44 ± 1.55	28.43 ± 1.16	> 500
*P. ornatum*	28.67 ± 0.494	38.22 ± 0.99	54.74 ± 0.62	428 ± 7
Standard Herbal	45.87 ± 0.546	65.49 ± 1.49	87.30 ± 0.67	151 ± 3

### α-Glucosidase inhibition

**Fig. 5:**
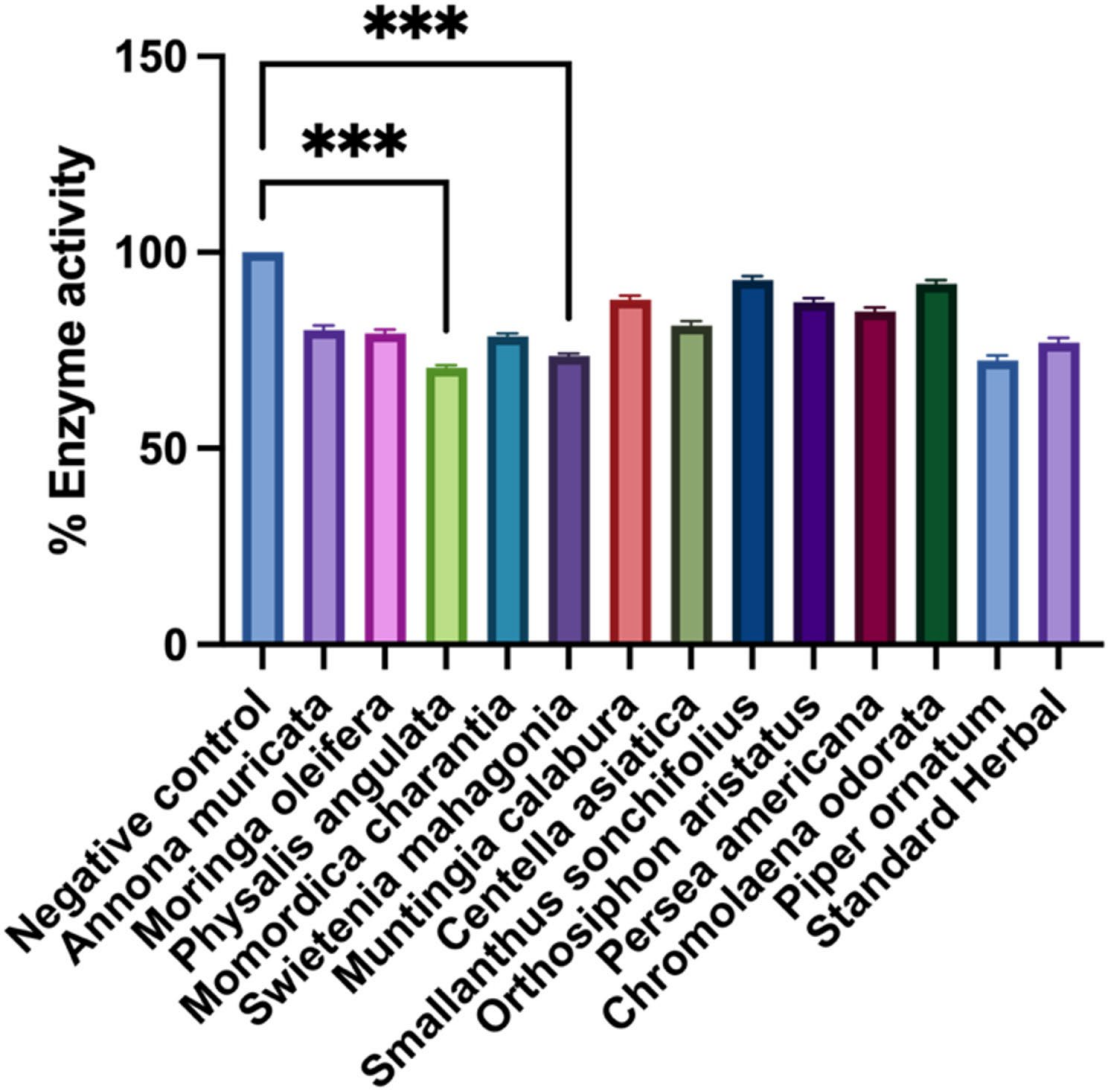
α-glucosidase % inhibition in samples treated with 125 µg/ml. The data are expressed as the mean ± SD. Asterisks (*) denotes significantly different (*P* < 0.001) from the negative control (Blank)

Table 3 presents the α-glucosidase inhibition data. Only *P. angulata* (52.10%) and the Standard Herbal comparator (52.68%) achieved > 50% inhibition at 500 µg/mL, with IC₅₀ values of 438 ± 8 µg/mL and 454 ± 3 µg/mL, respectively. Other extracts demonstrated lower inhibition, indicating IC₅₀ values above 500 µg/mL. At the lowest tested concentration (125 µg/mL), *P. angulata*, *P. ornatum*, and *S. mahagoni* showed significantly greater inhibition compared to controls (*p* < 0.001) (Fig. 5).

**Table 3:** Inhibition of α-glucosidase activity by different extracts at various concentrations

Medicinal plants	Percentage of enzyme inhibition at various concentration			Calculated IC_50_ (µg/mL)
	125 µg/mL	250 µg/mL	500 µg/mL	
*A. muricata*	19.79 ± 1.155	27.59 ± 0.09	45.74 ± 1.11	> 500
*M. oleifera*	20.74 ± 1.000	28.43 ± 1.56	33.43 ± 1.16	> 500
*P. angulata*	29.39 ± 0.582	41.18 ± 1.20	52.10 ± 2.95	438 ± 8
*M. charantia*	21.39 ± 0.645	27.44 ± 1.17	35.35 ± 1.21	> 500
*S. mahagoni*	26.39 ± 0.607	31.71 ± 1.69	38.12 ± 1.08	> 500
*M. calabura*	12.05 ± 0.993	16.90 ± 1.14	26.38 ± 1.69	> 500
*C. asiatica*	18.73 ± 1.155	28.39 ± 1.19	20.91 ± 2.86	> 500
*S. sonchifolius*	7.05 ± 0.973	10.12 ± 1.09	13.89 ± 1.80	> 500
*O. aristatus*	12.72 ± 1.005	19.24 ± 1.73	17.72 ± 1.24	> 500
*P. americana*	15.05 ± 1.003	27.76 ± 1.55	9.12 ± 1.75	> 500
*C. odorata*	8.07 ± 0.977	10.69 ± 1.20	22.32 ± 1.67	> 500
*P. ornatum*	27.51 ± 1.232	28.40 ± 2.48	20.37 ± 2.39	> 500
Standard Herbal	23.01 ± 1.175	33.38 ± 1.16	52.68 ± 1.70	454 ± 3

### DPPH radical scavenging activity

Based on high phenolic and flavonoid contents, six extracts were selected for further antioxidant analysis. Among these, *S. mahagoni* displayed the most potent antioxidant activity (IC₅₀ = 137.5 ± 11.7 µg/mL), followed closely by *P. americana* and *M. calabura* (Table 4).

**Table 4:** DPPH radical scavenging activity of the six selected plants

Medical Plants	Concentration (µg/mL)						IC_50_ (µg/mL)
	31.25	62.50	125	250	500	1000	
*S. mahagoni*	19.27 ± 4.56	32.03 ± 2.10	51.01 ± 1.94	81.45 ± 0.48	93.30 ± 0.91	94.14± 0.38	137.5 ± 11.7
*P. americana*	14.24 ± 3.91	25.24 ± 0.65	46.88 ± 3.55	65.69 ± 2.72	84.74 ± 1.17	89.03± 1.86	143.5 ± 15.6
*A. muricata*	12.97 ± 3.01	23.33 ± 2.56	37.43 ± 0.86	61.26 ± 2.60	90.51 ± 0.90	91.89± 0.91	201.3 ± 31.6
*M. calabura*	15.46 ± 7.57	22.70 ± 13.17	44.17 ± 11.46	83.28 ± 6.27	91.58 ± 1.35	92.88± 0.51	146.1 ± 5.6
*P. ornatum*	13.78 ± 2.11	24.81 ± 2.15	38.80 ± 1.99	64.39 ± 1.64	86.64 ± 1.29	92.31± 0.90	188.8 ± 17.9
*P. angulata*	4.24 ± 1.21	7.50 ± 0.70	13.19 ± 1.26	20.50 ± 2.62	48.22 ± 0.70	81.52± 0.72	543.1 ± 105.1

### Principal-component analysis (PCA)

**Fig. 6:**
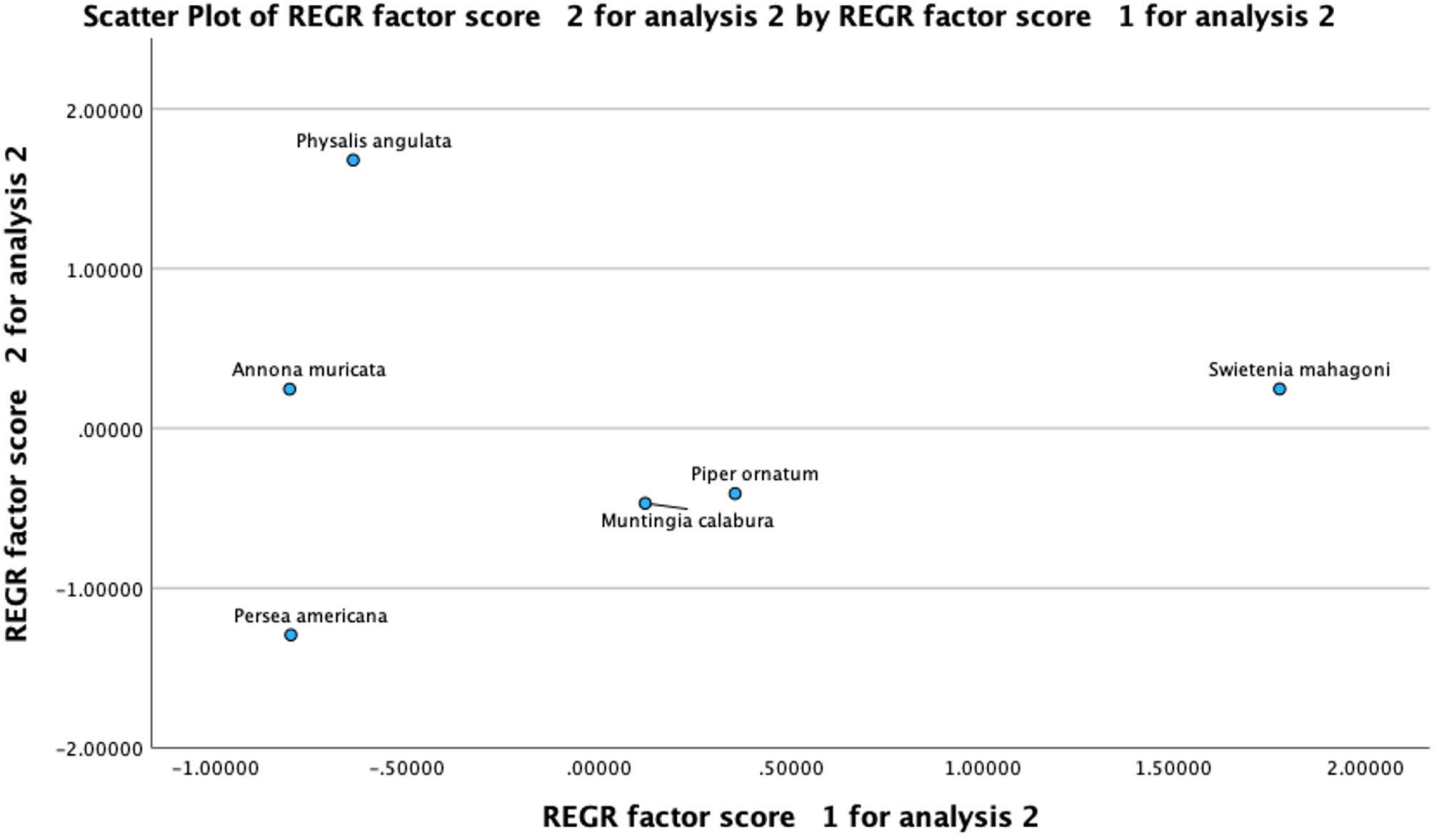
PCA scatter plot of principal-component regression scores for medicinal plants based on phytochemical and bioactivity variables

Principal-component analysis (PCA) was employed to elucidate relationships among phytochemical content (TPC and TFC) and biological activities (% enzyme inhibition and DPPH radical scavenging IC₅₀ values) of the extracts. Three principal components (PCs) emerged with eigenvalues exceeding 1.0, cumulatively explaining 95.72% of the total variance. Individually, PC-1 accounted for 36.94%, PC-2 for 36.11%, and PC-3 for 22.67% of the total variance. The Kaiser–Meyer–Olkin (KMO) measure of sampling adequacy was 0.30, indicative of exploratory adequacy given the small dataset, while Bartlett’s test of sphericity yielded a χ² of 12.0 (df = 10, *p* = 0.28), suggesting adequate intercorrelation among variables to justify PCA. The rotated component matrix (Table 5) indicates distinct bioactivity profiles. PC-1 strongly correlated phenolic content with α-amylase inhibition, highlighting *S. mahagoni*. PC-2 linked potent α-glucosidase inhibition with weaker antioxidant activity, typified by *P. angulata*. PC-3 reflected flavonoid content independently, with *A. muricata* dominating. Figure 6 visually summarizes these clusters.

**Table 5:** Rotated component matrix and communalities

Variable (z-score)	PC-1	PC-2	PC-3	Communality (h²)
% α-Amylase inhibition (500 µg/mL)	**0.991**	0.045	0.085	0.983
Total phenolic content (TPC)	**0.841**	–0.472	–0.102	0.992
% α-Glucosidase inhibition (500 µg/mL)	0.268	**0.93**	0.175	0.927
DPPH IC₅₀ (µg/mL) †	–0.378	**0.845**	–0.301	0.907
Total flavonoid content (TFC)	0.044	–0.056	**0.979**	0.978

(absolute loadings ≥ 0.50 are shown in bold)

† Higher IC₅₀ = weaker antioxidant potency

## Discussion

This study comparatively analyzed total flavonoid content (TFC), total phenolic content (TPC), enzyme inhibition activities, and DPPH scavenging capacities of selected medicinal plant extracts. Among these, *S.mahagoni*, *A.muricata*, and *P.americana* showed notably high TFC and TPC, correlating well with their enzyme inhibitory activities and antioxidant potentials. Specifically, *S. mahagoni* exhibited the strongest α-amylase inhibition, while *P. angulata* was the most potent α-glucosidase inhibitor. At a concentration of 125 µg/mL, *S. mahagoni* demonstrated α-amylase inhibition comparable to standard drugs, significantly outperforming the negative control along with *M.calabura*, *S.sonchifolius*, and *O.aristatus* (*p* < 0.001).

The α-amylase inhibition is an important therapeutic target for managing type II diabetes mellitus. The activity of this enzyme in the small intestine is linked to increased postprandial glucose levels. By inhibiting pancreatic α-amylase, the digestion of oligosaccharides into absorbable monosaccharides is delayed, thereby reducing postprandial blood glucose levels [12, 22]. Inhibitors of α-amylase are valuable therapeutic agents as they reduce glucose absorption without inducing hyperinsulinemia, particularly in type 2 diabetes [6, 23]. Likewise, α-glucosidase inhibitors act by slowing down the final hydrolysis step of carbohydrates in the small intestine, diminishing postprandial glucose absorption and subsequently lowering blood glucose concentrations [6, 9, 20]. Our findings showed significant α-glucosidase inhibitory activity from *P. angulata*, *P. ornatum*, and *S. mahagoni* at the tested dose of 125 µg/mL. Furthermore, dual enzyme inhibitory activity was observed in several plants, aligning with prior ethnopharmacological studies [24].

Traditionally, α-amylase and α-glucosidase inhibitory activities have been attributed to phenolics and flavonoids, similar to the action of synthetic drugs like acarbose [7, 25–28]. The inhibitory activity of phenolic compounds, such as phenolic acids and flavonoids, on α-amylase and α-glucosidase enzymes are associated with their structural properties [29, 30]. These compounds can interact with enzymes or reaction substrates, binding covalently to α-amylase. This alters the enzyme’s activity through the formation of quinones or lactones that react with nucleophilic groups on the enzyme molecules [31, 32]. Furthermore, the total phenolic and flavonoid contents are strong indicators of free radical scavenging activity. Phenolic compounds, which are major representatives of secondary plant metabolites, are among the most common natural antioxidants and can be classified into different subgroups based on their structural properties, including the flavonoid group. Higher levels of these compounds generally correlate with greater antioxidant potential, as they can donate hydrogen atoms or electrons to neutralize free radicals. The antioxidant potency of these compounds has been extensively documented in various publications [10, 25–28, 33–37].

The PCA scatter plot visually identify *S. mahagoni* as a phenolic-rich extract with dual antioxidant and α-amylase inhibitory activities, positioning it as a prime candidate for further pharmacological investigation. These observations corroborate prior studies by Syame et al. which reported the strong antioxidant activity of *S. mahagoni* highlighting the substantial antioxidant and antidiabetic potentials of *S. mahagoni*, attributed to its diverse phytochemicals including phenolics, flavonoids, alkaloids, and limonoids [38]. *S. mahagoni* has been widely reported for its antidiabetic effects. A recent review on the use of *S. mahagoni* reported that the seed, bark, or leaf extracts of *S. mahagoni* exhibit antidiabetic activity comparable to that of synthetic drugs with relatively mild toxic effects. The proposed hypoglycemic mechanisms include reducing blood glucose levels, restoring liver function and pancreatic β-cell function, possibly blocking the function of epinephrine, inhibiting α-amylase and β-glucosidase, and providing antioxidant and antihyperlipidemic effects. The phytochemical compounds of *S. mahagoni* include phenolics (including flavonoids such as swietemacrophyllanin, catechin, epicatechin, and tannins), triterpenoids, and tetranortriterpenoids (limonoids: mahonin, secomoganin, swietmanin, swiemahogin, swietenine, and swietenolide), saponins, and alkaloids known for their antidiabetic properties [38, 39].

Our PCA analysis also reveals an orthogonal activity archetype in *P. angulata*, which clusters strongly on PC-2. Although characterized by only moderate phenolic/flavonoid levels, *P. angulata* exhibits pronounced α-glucosidase inhibition (IC₅₀ = 438 µg/mL). Previous study reported that P.angulata extract show concentration-dependent α-amylase and α-glucosidase inhibition in vitro and demonstrate hypoglycemic effects in streptozotocin-induced diabetic animal models [40, 41]. These divergent yet complementary activity patterns suggest a combination strategy of *S. mahagoni* for its phenolic-driven antioxidant–α-amylase targeting, paired with *P. angulata* its glucosidase inhibition.

This study represents the initial phase in systematically identifying promising medicinal plants for diabetes management, providing crucial insights into optimal plant selection and combination strategies. However, limitations, such as the exploratory nature due to a small sample size (KMO = 0.30), underscore the preliminary nature of our findings. Subsequent research should include comprehensive toxicity and efficacy evaluations, employing both cytotoxicity assays and in vivo studies, to validate safety and therapeutic effectiveness. Ultimately, this approach aims to bridge traditional medicinal knowledge with modern pharmacological methods, fostering affordable, culturally acceptable, and sustainable therapeutic solutions for diabetes.

## Conclusions

This study provides the first integrated phytochemical–bioactivity map for twelve Sundanese antidiabetic plants. Among the extracts, *S.mahagoni* combined the highest phenolic content with the strongest α-amylase inhibition and the most potent antioxidant activity. Principal-component analysis confirmed two orthogonal bioactivity clusters namely phenolic–amylase and glucosidase–antioxidant axis. Flavonoid abundance varied independently of enzyme inhibition, illustrating that high TFC alone is not predictive of antidiabetic efficacy. These multivariate trends validate ethnomedicinal knowledge and highlight *S. mahagoni* and *P. angulata* as promising candidates for further pharmacological studies. The findings emphasize the need for further research to isolate and identify these compounds. Moreover, the localized focus on Sundanese medicinal plants, combined with rigorous experimental approaches, ensures that the results are both culturally relevant and globally applicable. This study not only highlights the potential of these plants for natural antidiabetic therapies but also bridges the gap between traditional medicine and modern pharmacological research, paving the way for sustainable and community-based healthcare solutions.

## Supplementary Information

Supplementary Material 1.

## Data Availability

All data supporting the findings of this study are included in this article and its Supplementary Material.
