# Supplementary material for: Alpha-amylase and Alpha-glucosidase enzymes inhibition and antioxidant potential of selected medicinal plants used as anti-diabetes by Sundanese community in West Java, Indonesia
Source: BMC Complement Med Ther. 2025 Nov 14;25:426. doi: 10.1186/s12906-025-05144-x (PMC12619263; doi:10.1186/s12906-025-05144-x)
Supplement: Supplementary file 1 — Supplementary Material 1. [file 12906_2025_5144_MOESM1_ESM.pdf]

KEMENTERIAN RISET, TEKNOLOGI DAN PENDIDIKAN TINGGI
UNIVERSITAS PADJADJARAN FAKULTAS KEDOKTERAN
KOMISI ETIK PENELITIAN KESEHATAN
HEALTH RESEARCH ETHICS COMMITTEE
Jl. Prof. Eyckman No. 38 Bandung 40161
Telp. & Fax. 022-2038697 email: kepk.fk.unpad@gmail.com, website : kepk.fk.unpad.ac.id

No. Reg.: 0617060773

PERSETUJUAN ETIK
ETHICAL APPROVAL

No: 750/UN6.C.10/PN/2017

Komisi Etik Penelitian Kesehatan Fakultas Kedokteran Universitas Padjadjaran Bandung, dalam upaya melindungi hak asasi dan kesejahteraan subjek penelitian kesehatan dan menjamin bahwa penelitian yang menggunakan formulir survei/registrasi/surveilens/Epidemiologi/Humaniora/Sosial Budaya/Bahan Biologi Tersimpan/Sel Punca dan non klinis lainnya berjalan dengan memperhatikan implikasi etik, hukum, sosial dan non klinis lainnya yang berlaku, telah mengkaji dengan teliti proposal penelitian berjudul:

The Health Research Ethics Committee Faculty of Medicine Universitas Padjadjaran Bandung, in order to protect the rights and welfare of the health research subject, and to guaranty that the research using survey questionnaire/registry/surveillance/epidemiology/humaniora/social-cultural/archived biological materials/stem cell/other non clinical materials, will carried out according to ethical, legal, social implications and other applicable regulations, has been throughly reviewed the proposal entitled:

"UBAR KAMPUNG *IN THE SUNDANESE REGION OF WEST JAVA: A COMPLEMENTARY ETHNOPHARMACOLOGICAL STUDY OF THE KNOWLEDGE AND USE OF MEDICINAL, AROMATIC AND COSMETIC (MAC) PLANTS FOR THE TREATMENT OF DIABETES MELLITUS IN INDONESIA.*"

Nama Peneliti Utama<br>*Principal Researcher*	:	Raden Maya Febriyanti
Pembimbing/Peneliti Lain<br>*Supervisor/Other Researcher*	:	Prof. DR. L. J. Slikkerveer
Nama Institusi<br>*Institution*	:	LEAD Programme<br>Faculty Of Science Leiden University

proposal tersebut dapat disetujui pelaksanaannya.
hereby declare that the proposal is approved.

Ditetapkan di : Bandung
Issued in
Tanggal : 03-07-2017
Date

Ketua
Chairman

Prof. Dr. Firman F. Wirakusumah, dr.,SpOG-K
NIP. 19480115 197302 1 001

Keterangan/*notes:*
Persetujuan etik ini berlaku selama satu tahun sejak tanggal ditetapkan.
This ethical clearance is effective for one year from the due date.
Pada akhir penelitian, laporan pelaksanaan penelitian harus diserahkan ke Komisi Etik Penelitian Kesehatan.
In the end of the research, progress and final summary report should be submitted to the Health Research Ethics Committee.
Jika ada perubahan atau penyimpangan protokol dan/atau perpanjangan penelitian, harus mengajukan kembali permohonan kajian etik penelitian.
If there be any protocol modification or deviation and/or extension of the study, the Principal Investigator is required to resubmit the protocol for approval.
Jika ada kejadian serius yang tidak diinginkan (KTD) harus segera dilaporkan ke Komisi Etik Penelitian Kesehatan.
If there are Serious Adverse Events (SAE) should be immediately reported to the Health Research Ethics Committee
